# Practical Notes on Diagnosis and Treatment

**Published:** 1907-10-26

**Authors:** 


### Practical Notes on Diagnosis and Treatment.

**Vomiting of Pregnancy.**

In a severe case the hypodermic injection of normal saline solution twice a day in combination with lavage of the stomach every morning has proved successful.

**Pneumothorax.**

In rare instances pneumothorax occurs without any underlying disease that can be detected. No doubt in such circumstances a suspicion of pulmonary tubercle is justified, but it seems quite certain that an occasional case occurs apart from this explanation.

**Typhoid Fever.**

It is said that the development of a yellow colour on the palms and soles may be noted as an early symptom of typhoid fever. Another observation in connection with the same disease is the absence of the superficial abdominal reflexes.

**Hysterical Eructations.**

Hysterical patients sometimes acquire the habit of "gulping" air into the œsophagus and stomach, and then bringing it up in frequent and noisy eructations. The application of the faradic current will usually put a stop to this symptom.

**Tympanites.**

When this condition exists in at all a severe form it may cause much embarrassment to the heart and respiration, and thus prejudice the patient's chance of recovery. An enema of 1 to 2 drachms of oil of turpentine in three ounces of thin starch is an excellent remedy.

**Uræmia.**

Headache and sleeplessness in uræmic patients can generally be removed by the hypodermic injection of morphine. The fear that morphine is dangerous in these circumstances in view of the renal lesion is not well-founded. In many cases of acute uræmia morphine is the best remedy.

**Multiple Neuritis and Mumps.**

As an exceptional event a multiple peripheral neuritis has been observed after an attack of mumps. Pains in the limbs, and tenderness of the muscles to pressure, have been followed in due course by paralysis of all the limbs.

**Pregnancy and Morbus Cordis.**

Though cardiac valvular disease when compensated ought not to be interposed as a bar to marriage and pregnancy, the special risks of the condition are increased in successive pregnancies. Hence the danger of rapidly succeeding pregnancies in these circumstances ought to be emphasised.

**High Arterial Tension.**

Mercury, even in a small dose, produces a greater effect in lowering arterial tension than more powerful purgatives given freely. A widespread opinion prevails that mercury ought not to be given to reduce tension when there is albuminuria; this is a great mistake.

**Pericarditis.**

Acute pericarditis may heal and leave no adhesions, and thus need not affect insurance risk. The prognosis, or at least the immediate prognosis, in pericarditis occurring during acute rheumatism, is almost invariably good. As a complication of renal disease, however, pericarditis is of most anxious significance.

**Sprains of the Ankle.**

A method of treatment strongly advocated in preference to the usual plan of rest and elevation is that of firmly strapping the part and encouraging the patient to go about as usual, at least after the first twenty-four hours. The strapping is applied so as to enclose the whole foot except the heel; it can be kept on until pain and swelling have subsided, after which, as a rule, no further treatment is required.

